# A prospective study using an individualized nomogram to predict the success rate of external cephalic version

**DOI:** 10.1038/s41598-022-16112-7

**Published:** 2022-07-12

**Authors:** Jing Lin, Wei Liu, Wei Gu, Ye Zhou

**Affiliations:** 1 International Peace Maternity and Child Health Hospital, School of Medicine, Shanghai Jiao Tong University, 910 Hengshan Road, Shanghai, 200030 China; 2 Shanghai Key Laboratory of Embryo Original Diseases, Shanghai, China; 3 Shanghai Municipal Key Clinical Specialty, Shanghai, China

**Keywords:** Health care, Medical research

## Abstract

To establish a clinical-based nomogram for predicting the success rate of external cephalic version (ECV) through a prospective study. This was a single-center prospective study that collected eligible breech pregnant women. 152 participants were enrolled in the training cohort, who received ECV procedures performed by a single operator. We used the training cohort to establish regression equations and prediction models. These variables include maternal factors (age, operation gestational age, pre-pregnancy BMI (Body Mass Index), operation BMI, BMI increase, multipara), ultrasound factors (fetal weight estimation, amniotic fluid index, placental location, type of breech presentation, spinal position), and anesthesia. Univariate and multivariable analyses were used to screen the factors affecting the success of ECV. A nomogram scoring model was established based on these factors. And C-index, DCA (Decision Curve Analysis) and calibration curve, Hosmer–Lemeshow test was used to verify the prediction effect of the model. Finally, 33 participants were enrolled in the testing cohort who received ECV with an unrestricted operator. We used C-index, DCA (decision curve analysis), and Hosmer–Lemeshow to verify the application value of the prediction model. The calibration curves and ROC curves of both the training cohort and testing cohort are plotted for internal and external validation of the model. The ECV success rate of the training cohort was 62.5%. Univariate analysis showed that the predictors related to the success rate of ECV were age, BMI increase value, AFI (amniotic fluid index), breech type, placental location, spinal position, anesthesia, and multipara. The prediction thresholds of the corresponding indexes were calculated according to the Youden index. Multivariate logistic regression analysis showed that BMI increase ≥ 3.85 kg/m^2^, AFI ≥ 10.6 cm, anesthesia, multipara, and non-anterior placenta were independent predictors of ECV success. Through the internal and external validation, it is confirmed that the model has a good calibration and prediction ability. Our nomogram has a good ability to predict the success rate of ECV.

## Introduction

The global cesarean section rate has increased from about 23% to 34% in recent ten years, in which abnormal fetal position is the third indication (about 17%)^1^. A breech presentation occurs in full-term pregnancies of about 3–4%^2^. For breech singletons, most women choose a cesarean section, possibly considering the high risk and complications of breech delivery^3^. In 2000, the Term Breech Trial was published, and recommended cesarean section is recommended for breech fetuses to reduce the risk of complications such as 5-min Apgar score, perinatal mortality, and delivery trauma^4^. But even in developed countries, c-section is still the leading cause of maternal mortality and postpartum incidence rate^5^. In recent years, efforts are being made to reduce the number of cesarean sections, in part by encouraging doctors to change their management practices. In 2006, the recommendations issued by ACOG (American College of Obstetricians and Gynecologists) reiterated the existing guidelines that obstetricians should provide and implement ECV (external cephalic version) as much as possible^6^. ECV provides a method to reduce cesarean section. The operation involves applying pressure to the pregnant woman's abdomen to roll the fetus forward or backward to achieve vertex presentation. Especially in resource-poor environments, women may not have access to medical services during childbirth, and they may not be able to perform a cesarean section or unsafe. ECV may be particularly important.

A series of cases reported that the incidence of complications of ECV was very low^7^. Fetal heart rate abnormalities (4.7%) are common, but these are usually transient and improve after completion or abandonment of surgery. The incidence of more serious complications is less than 1%, including emergency cesarean section, premature rupture of membranes, umbilical cord prolapse, infant femoral fracture, fetal-maternal bleeding, and stillbirth^8^. Although complications are rare, ECV should be attempted where an emergency cesarean section can be performed. For this reason, this study chose to perform ECV in the operating room, although this is not necessary^9^. A computer-based decision model proposes that ECV seems to be cost-effective as long as the success probability is greater than 32%^10^. In another study, Nassar^11^ assessed decision-making assistance to women with the full-term breech position. Women receiving decision-making assistance experienced less decision-making conflict, more knowledge, and higher decision-making satisfaction. The purpose of the decision aid is not to improve or reduce the intervention rate, but to support informed decision-making in line with personal values. Given the safety and low risk of ECV, we recommend that all women with a breech position near full-term should try ECV if there are no contraindications. The purpose of this study is to establish a simple and accurate scoring system for predicting the success rate of ECV. This model can be a useful tool in decision-making and can be applied to all women. Because it helps to predict the probability of personalized success, helps to translate the data of a large cohort into daily practice at the patient level, and contributes to joint decision-making at the individual level.

## Methods

### Sample size calculation

According to the literature, the incidence of breech position at baseline is estimated to be about 5%. According to the previous 200 ECV cases carried out by the operator, the success rate of ECV is estimated to be about 65%. The sample size calculation determined that 151 subjects were needed to prove that there was a 65% difference in ECV success rate (α = 0.05, power = 80%, one-sided). Considering that about 10% of patients withdrew and lost follow-up, 170 patients were continuously included in the training cohort.

### Participants

This was a single-center prospective study that recruited 205 eligible breech pregnant women. From November 1, 2019, to October 31, 2021, 170 breech pregnant women were enrolled in the study as the training cohort. They all received regular prenatal examinations and intended to deliver their babies in our hospital. The participants in the training cohort received a trial of ECV by a single operator with experience of more than 200 ECVs. After establishing the model, we continued to collect 35 pregnant women with intended ECV from November 1, 2021, to April 30, 2022, as the testing cohort. They received the ECV with an unrestricted operator. The performer of the versions was not aware of the data previously collected and thus was not influenced by them. All the participants provided written informed consent to participate in this study. The study protocol was approved by The Ethics Committee of the International Peace Maternal and Child Health Hospital (reference number (GKLW 2020-104). All methods were performed by the relevant guidelines and regulations. This study has been registered with the Chinese clinical trial registry (www.clinicaltrials.gov) under registration number ChiCTR1900027062 (date of registration: 30/10/2019).

### Study outcomes

The primary outcome was the success rate of ECV. The success of ECV is determined by the immediate results (conversion to vertex presentation) after the operation, not the fetal position at delivery. The secondary outcomes included mode of delivery, gestational age at delivery, and pregnancy outcomes (eg, postpartum hemorrhage, Apgar, neonatal weight, etc.).

### Inclusion and exclusion criteria

ECV is carried out according to the standardization agreement of the organization. Inclusion criteria: The procedure is suitable for all breech pregnant women requiring ECV in a singleton pregnancy, intact fetal membranes, non-cephalic presentation, and gestational age of 36 weeks or longer^12^. Exclusion criteria: Including placenta previa or placental abruption^13^, unexplained bleeding, contraindications to vaginal delivery, intrauterine growth restriction associated with abnormal umbilical artery doppler index, alloimmunization, maternal or fetal diseases requiring immediate delivery, and fetal or uterine malformations.

### Operation flow and termination signal

After obtaining informed consent, the subjects were required to fast for at least 6 h before the operation. ECV was required to be performed in the obstetric operating room in case of the need for an emergency cesarean section. Women were asked to empty their bladder and then underwent preoperative ultrasound to confirm fetal presentation, spinal position, fetal heart rate, fetal activity, placental location, and amniotic fluid index. According to the recommendations of the literature^14,15^, all patients received intravenous β-receptor-agonists (ritodrine hydrochloride) 20 min before ECV operation to relax the uterus and make the fetus more easily moved. The dose for intravenous infusion administration is as follows: initially, 50 µg/min, increased gradually according to the response by 50 µg/min every 10 min, usual dose: 0.25 mg/min. According to the wishes of the pregnant woman, she can choose whether to operate under anesthesia. Pregnant women under anesthesia were given 1% ropivacaine for spinal anesthesia. An experienced single obstetrician performs this procedure. The performer of the operation is unaware of the previously collected data and is therefore not affected by it. A maximum of four ECV attempts are allowed, with a 3–5 min’ rest between them to check the FHR and fetal position. During the ECV procedure, the health of the fetus is monitored by an ultrasound scan focusing on the fetal heartbeat and movement, the safety of the fetus is confirmed by an ultrasound scan 1 h after the procedure, and an NST (non-stress test) is performed for at least 20 min. Terminate the session in any of the following cases: (1) the fetus was successfully inverted to vertex, (2) after 30 min or more than four fetal operations, (3) the patient asked to stop the operation due to pain or any other reason, and (4) abnormal fetal heart rate was detected.

### Statistical analyses

SPSS statistical software version 21.0 (Armonk, NY: IBM Corp, https://www.ibm.com/products/spss-statistics) and R (www.rproject.org) version 3.1.2 were used in this study. The measurement data are expressed as mean ± standard deviation, and the independent sample t-test is used for statistical comparison. The enumeration data were expressed as a percentage and compared by the chi-square test. The best prediction critical value of continuous measurement data is determined by the maximum Youden index of the diagnostic test. Youden index is a method to evaluate the authenticity of a screening test, which is the sum of sensitivity and specificity minus 1. The larger the index, the better the effect of the screening experiment. Odds ratios (ORs) were calculated by logistic regression with stepwise input covariates. The odds ratios were expressed by 95% confidence interval (CI) to determine the influencing factors and evaluate the prediction effect. The significance level was 0.05 (two-tailed). A nomogram for predicting the ECV success rate was built using the R library “rms” package. We integrated all parameters that remained independent predictors of the Logistic model. The training cohort was used to establish the prediction model and conduct internal validation. The testing cohort was used for subsequent external validation. In addition to using the C-index, DCA, and Hosmer–Lemeshow test to evaluate the predictive value of the model, we also used calibration curves and ROC (receiver-operating characteristics) curves for both internal and external validation of the model.

### Ethics approval

The Ethics Committee of the International Peace Maternal and Child Health Hospital approved the study’s procedures (reference number: GKLW 2020-104). This study has been registered in the Chinese clinical trial registry (www.clinicaltrials.gov) (registration number ChiCTR1900027062). All participants provided written informed consent and the ethics committee approved the consent procedures.

## Result

### Characteristics of the patient

**Figure 1:**
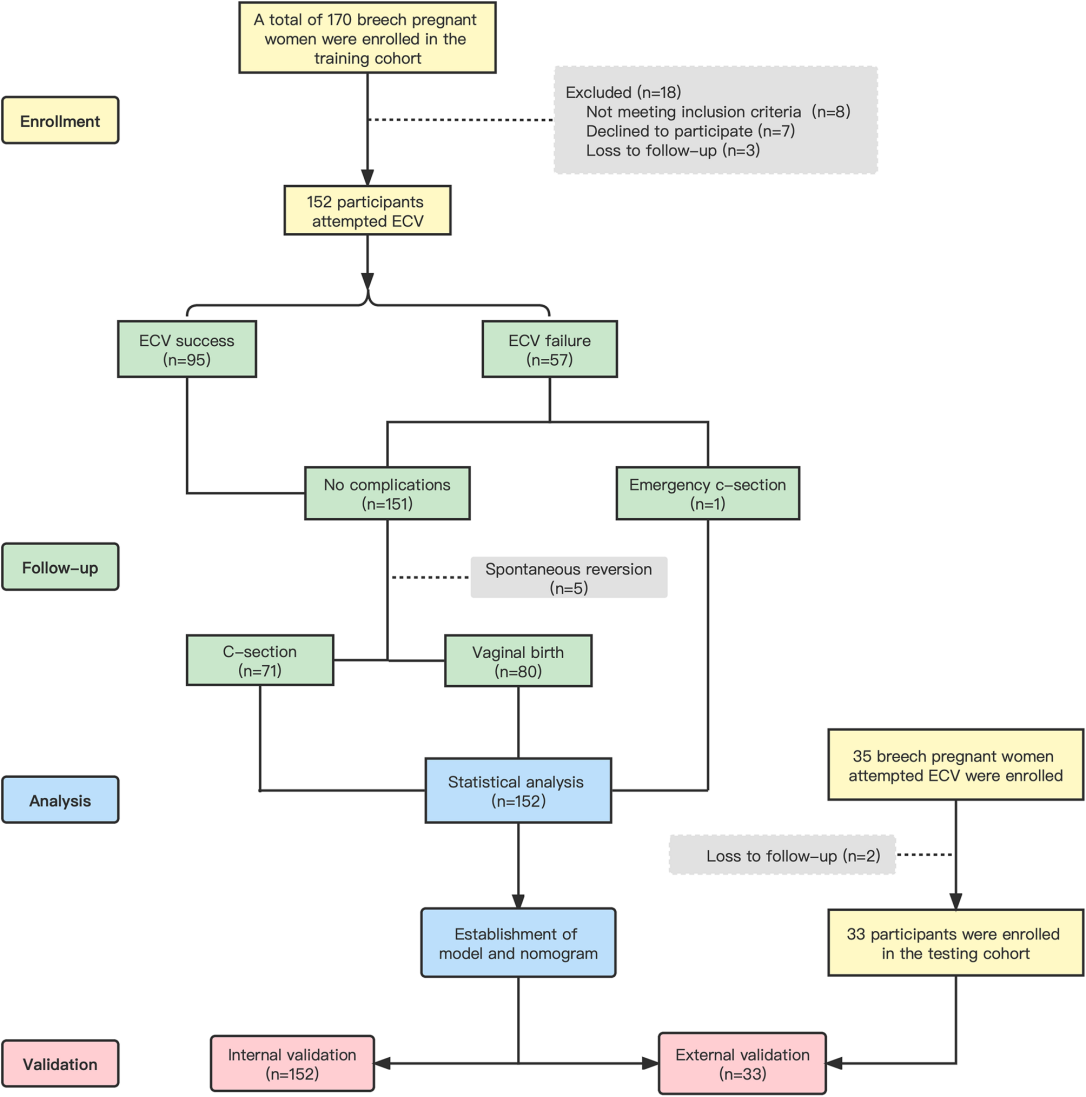
The flowchart of this study.

Among the 170 breech pregnant women recruited as the training cohort, 8 (4.7%) breech women did not meet the inclusion criteria of ECV. Among them, are 3 cases of premature rupture of membranes, 3 cases with placenta previa, 1 case with uterine malformation, and 1 case with unexplained vaginal bleeding. Of the 162 women considered eligible for ECV, 7 (4.3%) refused the operation, and 3 (1.9%) gave birth in another hospital or lost follow-up. Overall, 152 pregnant women were identified and opted for a trial of ECV by a single operator. The performer of the versions was not aware of the data previously collected and thus was not influenced by them. Among them, external inversion was successful in 95 cases and failed in 57 cases. After the model was established, we continued to recruit 35 pregnant women as the testing cohort. After excluding two lost subjects, 33 pregnant women were finally included. The flowchart is shown in Fig. 1.

The average age of the participants in the training cohort was 31.6 ± 3.7 years old. There were 79 multiparous women, accounting for 52%. And the gestational age at operation was 36.8 ± 0.5 weeks. The BMI before pregnancy was 20.8 ± 2.4 kg/m^2^. The BMI at operation was 25.7 ± 2.7 kg/m^2^. And the BMI increase value was 4.9 ± 1.5 kg/m^2^. The estimated fetal weight was 2992.9 ± 362.7 g and the amniotic fluid index was 13.0 ± 2.8 cm. The comparison results of baselines in two groups are shown in Table 1. There were significant differences in age, BMI increase value, amniotic fluid index, and multipara between the two groups (*P* < 0.005). The average age of the successful ECV group was elder, the BMI increase value during pregnancy was smaller, and the amniotic fluid index and the rate of multipara were higher.

**Table 1:** Demographic and clinical characteristics of the training cohort (N = 152).

	ECV		*P*
	Failure (n = 57)	Success (n = 95)	
Age (year)	30.5 ± 3.7	32.2 ± 3.7	0.006
Operation gestational age (weeks)	36.8 ± 0.5	36.8 ± 0.6	0.580
Pre-pregnancy BMI (kg/m2)	20.8 ± 2.5	20.8 ± 2.4	0.951
Operation BMI (kg/m2)	26.1 ± 2.6	25.5 ± 2.8	0.186
BMI increase (kg/m2)	5.3 ± 1.3	4.7 ± 1.5	0.018
Fetal weight estimation (g)	2941.8 ± 373.33	3023.4 ± 354.7	0.179
Amniotic fluid index (cm)	12.2 ± 2.8	13.5 ± 2.8	0.006
Multipara (%)	20 (35.1%)	59 (62.1%)	0.001

### The ECV success rate and screening of predictive factors

152 pregnant women in the training cohort underwent external inversion, of which 95 cases were successful and 57 cases failed. The success rate of ECV was 62.5%. The following objective indicators were included in this study: maternal factors (age, operation gestational age, pre-pregnancy BMI, operation BMI, BMI increase, multipara), ultrasound factors (fetal weight estimation, amniotic fluid index, placental location, type of breech presentation, spinal position), and whether anesthesia was analyzed by univariate analysis (Table 2). The results showed that the predictors related to the success rate of ECV were age (OR = 1.139, 95% CI 1.036–1.251), BMI increase(OR = 0.757, 95% CI 0.599–0.958), amniotic fluid index (OR = 1.018, 95% CI 1.005–1.032), breech type (OR = 0.286, 95% CI 0.111–0.737), placental location (OR = 2.438, 95% CI 1.636–3.635) spinal position (OR = 0.390, 95% CI 0.238–0.639), anesthesia (OR = 3.254, 95% CI 1.428–7.417) and multipara (OR = 3.032, 95% CI 1.530–6.008), while other factors were not associated with success rate.

**Table 2:** Univariate analysis of possible prediction factors for ECV success.

		ECV		*P* value		OR	95%CI
		Failure (n = 57)	Success (n = 95)	*P*	*P* for trend*		
Age (year)		30.5 ± 3.7	32.2 ± 3.7	0.006	–	1.139	1.036–1.251
Operation gestational age (weeks)		36.8 ± 0.5	36.8 ± 0.6	0.580	–	1.189	0.646–2.189
Fetal weight estimation (g)		2941.8 ± 373.33	3023.4 ± 354.7	0.179	–	1.001	1.000–1.002
Pre-pregnancy BMI (kg/m^2^)		20.8 ± 2.5	20.8 ± 2.4	0.951	–	0.996	0.869–1.140
Operation BMI (kg/m^2^)		26.1 ± 2.6	25.5 ± 2.8	0.186		0.920	0.813–1.041
BMI increase (kg/m^2^)		5.3 ± 1.3	4.7 ± 1.5	0.018	–	0.757	0.599–0.958
Multipara (%)	No	36 (49.3%)	37 (50.7%)	0.004	–	3.032	1.530–6.008
	Yes	21 (26.6%)	58 (73.4%)				
Type of breech (%)	Transverse	1 (8.3%)	11 (91.7%)	Ref	0.006	Ref	Ref
	Complete Breech	48 (37.8%)	79 (62.2%)	0.073		0.150	0.019–1.196
	Frank Breech	8 (61.5%)	5 (38.5%)	0.016		0.057	0.006–0.585
Placental position (%)	Anterior	34 (65.4%)	18 (34.6%)	Ref	< 0.001	Ref	Ref
	Lateral	8 (23.5%)	26 (76.5%)	< 0.001		6.139	2.311–16.306
	Posterior	14 (23.0%)	46 (76.7%)	< 0.001		6.206	2.714–14.194
	Fundus	1 (16.7%)	5 (83.3%)	0.048		9.444	1.024–87.110
Position of spine (%)	Transverse	1 (8.3%)	11 (91.7%)	Ref	< 0.001	Ref	Ref
	Anterior	19 (30.6%)	43 (69.4%)	0.143		0.206	0.025–1.709
	Lateral	25 (39.1%)	39 (60.9%)	0.069		0.142	0.017–1.167
	Posterior	12 (85.7%)	2 (14.3%)	0.001		0.015	0.001–0.191
Anesthesia (%)	No	48 (44.9%)	59 (55.1%)	0.004	–	3.254	1.428–7.417
	Yes	9 (20.0%)	36 (80.0%)				
Amniotic fluid index (cm)		12.2 ± 2.7	13.5 ± 2.8	0.006	–	1.018	1.005–1.032

Measurement data is expressed by mean = SD. Count data is expressed by number (%).

*P for trend based on the Mantel–haenszel Chi-squared test for trends was used to test the outcome effect significance across different categories.

### Determining prediction thresholds for quantitative indicators and prediction categories for qualitative indicators

**Figure 2:**
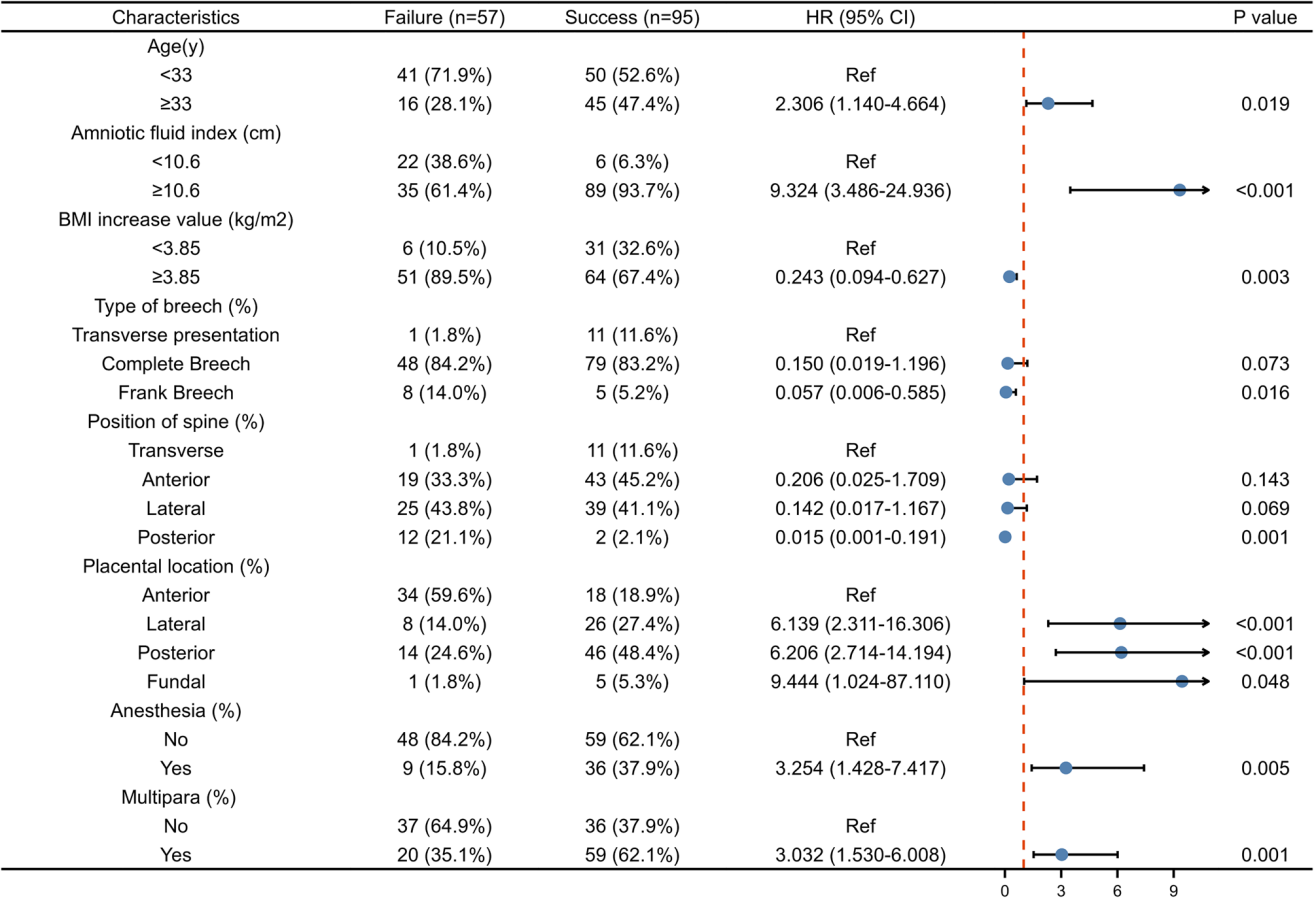
The forest plot of univariate analysis for predictive factors. CI indicates the confident interval.

After determining the predictors of ECV success (including 3 quantitative indicators and 5 qualitative indicators), a prediction model and scoring system were established. We first use the Youden index to determine the predictive thresholds of the above three quantitative indicators. Through calculation, we obtained the predicted thresholds of the following indicators: age was 33 years, the amniotic fluid index was 10.6 cm, and BMI increase was 3.85 kg/m^2^. For qualitative metrics, it is necessary to identify the categories that have the greatest impact on success rates. Among the breech presentation types, Frank breech had the lowest success rate (38.5%), and there was no statistical difference between the other two types. Among the spine positions, the success rate of the posterior spine was significantly lower than that of other positions (14.3%). Among placental locations, only the anterior wall placenta affected the ECV success rate (34.6%), and there was no significant difference among other locations. In addition, the success rate of ECV in both anesthesia and multiparous women was higher, 80.0% and 73.4%, respectively. The forest plot of univariate analysis for these risk factors were shown in Fig. 2.

### Determination of independent predictors and establishment of regression model

All the above predictors were put into multivariate logistic regression analysis (Table 3). The results showed that there were five independent factors affecting the success rate of ECV. BMI increase ≥ 3.85 kg/m^2^ (OR = 0.266, 95% CI 0.083–0.849), AFI ≥ 10.6 cm (OR = 9.191, 95% CI 2.823–29.924), anesthesia (OR = 4.264, 95% CI 1.512–12.027), multipara (OR = 2.688, 95% CI 1.077–6.709), non-anterior placenta (OR = 0.390, 95% CI 0.238–0.639). Based on these factors, we established a prediction model for ECV success rate.

**Table 3:** Multivariable logistic regression analysis of predictive factors for ECV success.

	B	Standard Error	Wald	*P*	OR	95% confidence interval	
						Lower	Upper
BMI increase ≥ 3.85 (kg/m2)	− 1.324	0.592	5.003	0.025	0.266	0.083	0.849
Amniotic fluid index ≥ 10.6 (cm)	2.218	0.602	13.566	< 0.001	9.191	2.823	29.924
Age ≥ 33 (years)	0.665	0.486	1.875	0.171	1.945	0.751	5.038
Anesthesia	1.450	0.529	7.512	0.006	4.264	1.512	12.027
Multipara	0.989	0.467	4.486	0.034	2.688	1.077	6.709
Non-anterior placenta	0.934	0.457	4.183	0.041	2.545	1.040	6.231
Posterior spine	− 1.642	0.884	3.445	0.063	0.194	0.034	1.096
Frank Breech	0.281	0.817	0.118	0.731	1.324	0.267	6.568
Constant	− 4.291	1.903	5.085	0.024	0.014		

### Establishment of the nomogram

**Figure 3:**
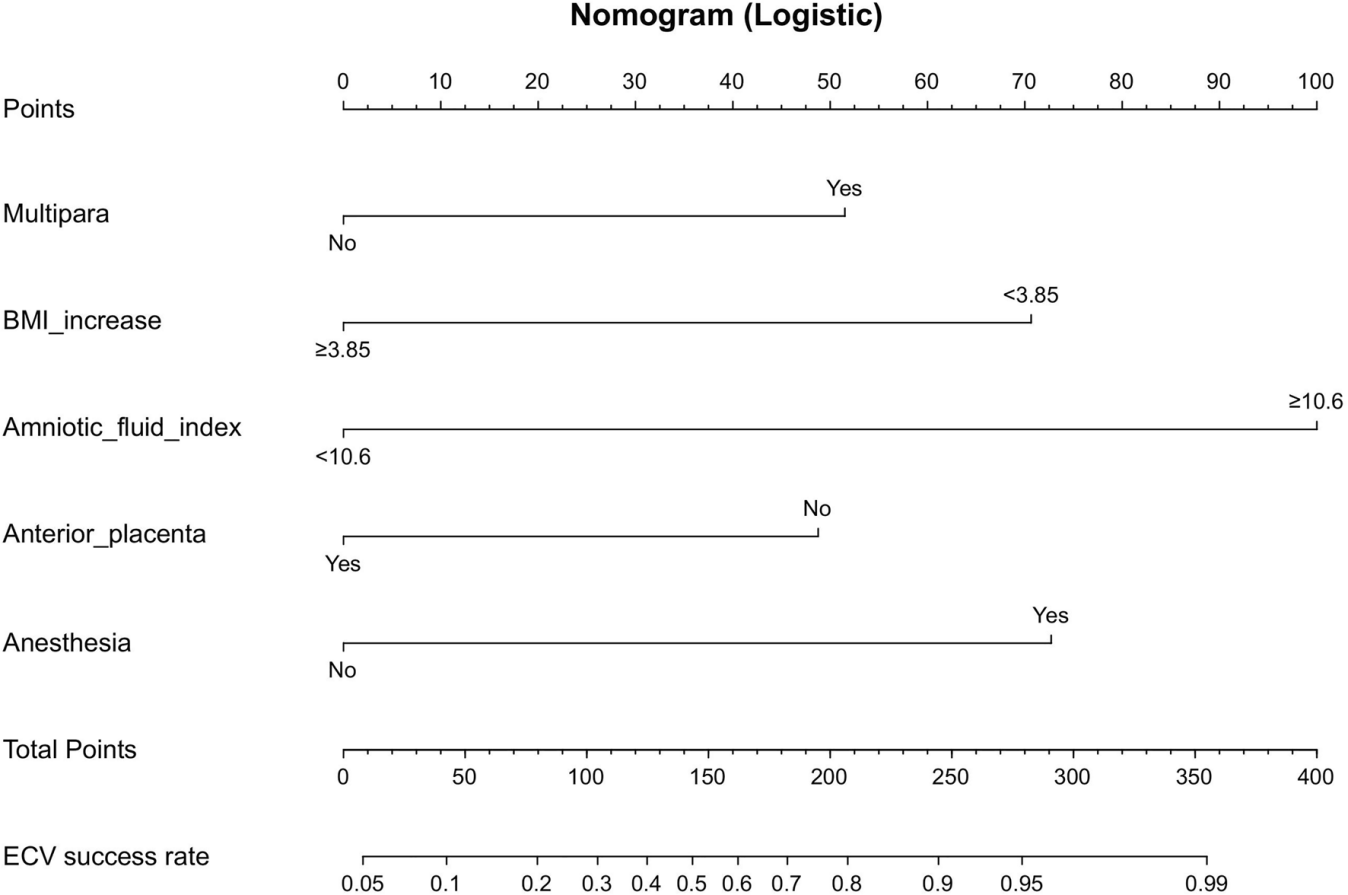
Nomogram for predicting ECV success rate. To calculate the success probability of ECV, draw a vertical line on the axis corresponding to each predictor until it reaches the line marked "point" at the top. Add up the points of all predictors and draw a line down the axis marked "total points" until it intersects the lower line showing the probability of ECV success.

**Figure 4:**
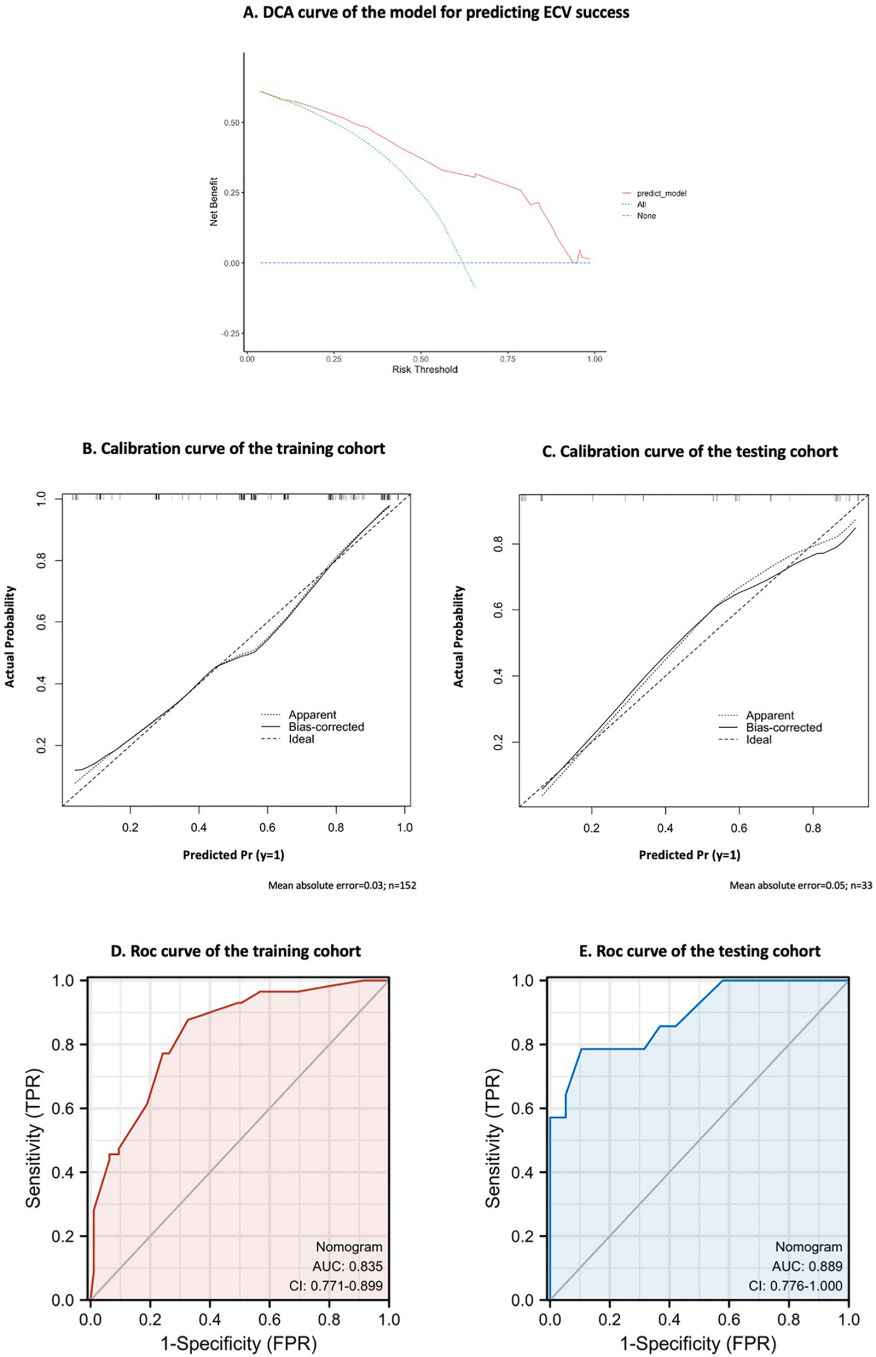
(**A**) Decision curve analysis (DCA) curve of the model for predicting ECV success. The y-axis represents the net income, the x-axis represents the threshold probability, and the red line represents the model. The blue line indicates that no pregnant women are assumed to have ECV, the green line indicates that all pregnant women are assumed to have ECV, and the red line indicates the results of the decision support model. The final DCA shows that if the threshold probability is between 10 and 90%, the strategy based on nomogram to predict the success rate of ECV in this study produces better net benefits than the "all ECV" and "no ECV" modes. In this range, the prediction effect of the nomogram is the best. (**B**, **C**) Calibration curves of the internal and external cohort. Nomogram-predicted probability of ECV success is plotted on the x-axis; actual probability of ECV success is plotted on the y-axis. The diagonal dotted line represents a perfect prediction by an ideal model. The solid line represents the performance of the nomogram. The closer this line is to the diagonal dotted line, the better the prediction. (**D**, **E**) ROC curves of the internal and external cohort. The prediction model built by a stepwise multivariable logistic analysis included five variables: BMI increase value, placental location, anesthesia, multipara, and amniotic fluid.

Based on these factors, we used the training cohort to establish a prediction model, which was given in the form of a nomogram scoring system (Fig. 3). The C-index was 0.841 and the Hosmer–Lemeshow test for the evaluation of calibration showed that the Chi-square value was 3.05 (*P* = 0.931 > 0.05) of the predictive model. We plotted a decision curve analysis (DCA) curve to estimate the potential of the clinical utility of the nomogram (Fig. 4A). The DCA curve indicated the net benefit of the nomogram was higher with the probability threshold ranging from 10 to 90%.

### Internal and external validation of the prediction model

We used calibration curves and ROC curves for both internal and external validation of the model. The calibration curve of the training cohort was shown in Fig. 4B and the calibration curve of the testing cohort was shown in Fig. 4C. The calibration curve of the nomogram for the prediction of ECV success was proven to be in good agreement. As shown, a perfect correlation between nomogram prediction and observed outcomes demonstrated great reliability of the nomogram. The ROC curve of the training cohort was shown in Fig. 4D, and the area under the curve (AUC) was 0.835 (95%CI 0.771–0.899). The ROC curve of the testing cohort was shown in Fig. 4E, and its AUC was 0.889 (95%CI 0.776–1.000).

### Adverse events and pregnancy outcomes of participants

In the process of external inversion, only one case in the failure group had fetal heart rate deceleration, which lasted for 60–80 bpm and did not recover. Therefore, an emergency cesarean section was performed. The other 151 cases had no adverse events or complications. Among 57 pregnant women in the ECV failure group, 3 cases had vaginal delivery after spontaneous inversion to a cephalic position. The other 54 cases were delivered by cesarean section, and the cesarean section rate was 94.7%. Among 95 pregnant women with ECV success, 77 had a vaginal delivery (including 75 cases of spontaneous delivery and 2 cases of forceps due to fetal distress). There were 18 pregnant women with successful ECV who were finally delivered by cesarean section. Among them, 2 cases underwent c-section after spontaneous inverted to shoulder presentation, 11 cases were fetal distress, 3 cases were macrosomia, 1 case had cephalopelvic disproportion and 1 case failed to induce labor. The pregnancy outcomes of the two groups are shown in Table 4. The results showed that the successful ECV group had a lower cesarean section rate, a later gestational age of delivery, a longer ECV-to-delivery interval, and higher neonatal birth weight. There were no significant differences in spontaneous inversion rate, postpartum hemorrhage volume and Apgar score between the two groups.

**Table 4:** Comparison of birth outcomes in the study population.

		ECV		*P*
		Failure(n = 57)	Success(n = 95)	
Delivery gestational age (weeks)		38.4 ± 0.7	39.3 ± 0.9	< 0.001
ECV to delivery interval (days)		7.0 ± 3.6	17.3 ± 7.8	0.008
Cesarean delivery rate (%)		54 (94.7%)	18 (18.9%)	< 0.001
Spontaneous version (%)		3 (5.4%)	2 (2.1%)	0.281
Postpartum hemorrhage (ml)		237.9 ± 65.5	246.1 ± 74.8	0.499
Presentations at delivery	Cephalic	3 (5.3%)	93 (97.8%)	< 0.001
	Breech	54 (94.7%)	1 (1.1%)	
	Transverse	0 (0%)	1 (1.1%)	
Apgar (score)		9.8 ± 0.7	9.9 ± 0.5	0.264
Neonatal weight (g)		3154.6 ± 357.1	3375.7 ± 382.1	0.001

## Discussion

The external cephalic version (ECV) is more and more widely advocated^5,16^. The trial can reduce the number of non-cephalic labor and cesarean delivery^17^, thereby reducing complications of breech delivery and maternal-infant incidence rate associated with cesarean section^18^. This is particularly important in a scarless uterus, as avoiding the first cesarean section reduces the number of repeat cesarean sections and the risk of surgical trauma associated with uterine adhesions, abnormal placental adhesions^19^, and uterine rupture^20^. ECV is a safe and effective procedure to reduce the cesarean section rate of breech pregnant women. The success rate of ECV surgery in this study was 62.5%, which was higher than 47.0–50.9%^20,21^ reported in international literature. This difference may be attributed to the extensive use of uterine contraction inhibitors and the rich experience of operators. As reported in this study, ECV has higher overall safety and fewer complications^22,23^. Our study found that 94.7% of pregnant women who failed in ECV still maintained breech positions before delivery, and all of them chose cesarean section. Routine use of cesarean section for breech presentation is widespread. A retrospective cross-sectional study of 109,736 pregnancies in 2016 found that the rate of single breech cesarean section in China was high, ranging from 83.06 to 98.62%^24^. Compared with planned vaginal delivery, the planned cesarean delivery reduced the incidence rate of perinatal or neonatal deaths and composite outcomes of death or severe neonatal morbidity^31^. In this study, all breech pregnant women chose a cesarean section and refused vaginal trial delivery, possibly considering the high risk and complications of breech delivery.

A more reliable and personalized prediction of successful ECV will help to provide women with advice on ECV attempts and improve individualized care and joint decision-making. Due to the different important variables included in the model, the performance of the model may be limited. Therefore, the purpose of this study is to determine which combination of important variables in daily clinical practice can best predict the success of ECV. Many factors are considered to affect the success rate of ECV, such as parity^31^, transverse spine, amniotic fluid index, posterior placenta, low BMI, and palpable fetal head. Objective indicators were included as predictors in this study to build a predictive model to improve clinical applicability. In this study, the univariate analysis found that maternal factors (age, multipara, BMI increase), ultrasound factors (amniotic fluid index, placental location, breech presentation type, spinal position), and anesthesia were associated with ECV success rates. The multivariate regression model found that BMI increase ≥ 3.85 kg/m^2^, AFI ≥ 10.6 cm, anesthesia, multiparity, and non-anterior placenta were independent influencing factors of ECV success.

In previous studies, multiparity was identified as the main maternal factor associated with the success rate of ECV^25,26^. It was well established that increased parity is associated with ECV success^27^. This may be because the abdomen of multiparous women is relatively loose, which is conducive to the implementation of external inversion. The results of this study are consistent with the above studies. The effect of age on ECV may be related to the proportion of multiparous mothers. Univariate found that age was positively associated with ECV success rate, but after adding parity, this association disappeared in multivariate analysis. A meta-analysis by Kok et al.^25^ showed that maternal weight below 65 kg was a significant predictor of ECV success. We believe that BMI provides a better estimate of the maternal abdominal wall because it considers both height and weight. BMI has been studied as a factor affecting the success rate of ECV^28^. Studies have shown that excessive weight gain during pregnancy is highly correlated with fat deposition in the abdominal region^29^. Numerous evidence shows that there is a high correlation between maternal abdominal subcutaneous fat thickness and BMI during pregnancy^30,31^. According to our results, the change in maternal BMI during pregnancy is an important determinant of ECV success. The likely reason is that the thicker abdominal wall complicates the manipulation of external inversion. We also demonstrated an independent association between increasing values of BMI and ECV outcome using multivariate logistic regression.

The first action of ECV operation is to lift the breech position from the maternal pelvis so that the fetus can rotate freely in the uterus. A large amount of amniotic fluid can increase the space for the fetus to turn around. Studies have proposed a positive correlation between amniotic fluid volume and success rate^27^. However, there are still disputes about the influence of amniotic fluid in the literature at present, mainly because the amniotic fluid factors in many studies are only described in the form of oligohydramnios or polyhydramnios, or different cut-off points are used to describe the relationship with ECV results, resulting in confusion. In order to avoid the above problems, this study uses an amniotic fluid index as a continuous variable to confirm the positive correlation between the amniotic fluid index and ECV success rate and then finds the threshold in the model as the prediction boundary through the Yoden index. We found that 106 as the threshold of the amniotic fluid index can effectively predict the success rate of ECV, and AFI ≥ 10.6 cm is an independent factor of ECV success. Many studies have reported the relationship between placental location and ECV results^32^. The anterior placenta was associated with less successful ECV, the posterior placenta had a higher ECV success rate, and the fundus and lateral placenta were not associated with ECV results. The results of this study are similar to the above results. Univariate analysis showed that the success rate of ECV in the anterior placenta is the lowest. Multivariate analysis showed that the anterior placenta was an independent factor affecting the success of ECV. As for the type of breech position, study^33^ found that the Frank breech position was negatively correlated with successful ECV, and the success rate of ECV in the complete breech position was higher, while the transverse position was most correlated with successful ECV. The results of this study were consistent with previous studies. The success rate of the transverse breech was the highest and that of the Frank breech was the lowest. Several studies^34^ have reported the relationship between fetal spinal position and ECV results. It was mostly found that the anterior or lateral spinal position could predict the success of ECV. This study found that the success rate of ECV in patients with the posterior spine was the lowest, which was consistent with the above results. However, multivariate analysis showed that breech type and spine position were not related to the success rate of ECV.

At present, routine use of local anesthesia is not recommended^35^, but repeated attempts for women who cannot tolerate ECV without analgesia can be considered. To minimize medical costs, our operation did not routinely use local analgesia during ECV. Although most women can tolerate ECV, they should be informed that this external force often makes women feel pain, causes contraction of abdominal wall muscles, and may limit the success rate of operation. A prospective study^36^ on ECV shows that this pain can undermine women's perception of surgery and reduce their chances of recommending the surgery to their peers, thus negatively affecting efforts to expand the adoption of the technology. Many studies^36,37^ have also suggested that pain is the most negative factor affecting pregnant women's use of ECV. One purpose of ECV analgesia is to reduce women's involuntary defense during operation, make it easier to move the fetus during ECV, which is conducive to the success of the operation, increase women's comfort, and encourage the wider use of the procedure^38,39^. Some studies have used local anesthesia in ECV, which reduces pain scores and improves success rate^40^. The results of this study showed that the use of anesthesia could significantly improve the success rate of ECV in both univariate analysis and multivariate regression (OR = 4.26, 95%CI 1.52–12.027).

Some literature^41^ proposed that the factors affecting the success rate of ECV include the height of the fetal presentation, palpation of the fetal head, uterine tension, etc. However, due to the subjective nature of these factors and the lack of a uniform measure, it is difficult to estimate their impact on the success rate. And these variables need to be collected during the ECV process, not during ECV consultation, so we do not think they should be included in the model. Our study developed a predictive score for ECV success rate as a simple tool that can be used for clinical decision-making. We found five independent predictors of ECV success: amniotic fluid index ≥ 10.6 cm, maternal BMI increase ≥ 3.85 kg/m^2^, non-anterior placenta, parity, and anesthesia.

Women who attempt ECV have an approximately 40% lower risk of cesarean section^2^. Providing information is the key to avoiding rejection, and it can still be improved. From a preference study^42^, we know that women are willing to accept treatments that may have side effects if they increase their chances of success. ECV success rates reported in the literature vary widely, and in addition to pain and concerns about adverse risks, uncertainty about success rates results in 76% of patients rejecting it. We believe that the three pillars of information about ECV are the high probability of success, the safety of the technique, and the rate of vaginal delivery after successful ECV. Having a successful predictive model can optimize information in a personalized way^43^. To improve the practicability of clinical practice, we propose a scoring system that allows individualized calculation of the success rate of ECV. We believe that our model is more useful and effective in clinical consultation. From a clinical point of view, the prediction model in our study can help clinicians use it as a decision-making tool and provide explanations for informed decision-making. We believe that the model should apply to all women because it helps to provide information for clinical practice, provide more knowledge for pregnant women, and provide an opportunity to improve the decision-making process.

### Advantages and limitations

Our research has several advantages. To the best of our knowledge, this study is the first prospective clinical study of ECV conducted by one operator. It is firmly believed that the experience of obstetricians or midwives is essential to improving the success rate of ECV^44,45^. Yun Kim^46^ developed a standardized learning curve guide for ECV and proposed that to achieve a 50% success rate, at least 57 ECV attempts are required, which emphasizes the potential importance of operator experience as a potential interference or predictor of successful ECV. Unfortunately, this variable is not controlled in many studies, which will lead to a series of deviations from the technical or experience differences of operators. Our study was conducted in a tertiary specialized hospital, and all subjects were completed by an experienced doctor. In addition, by focusing on the ECV performed by a single operator, we can offset any deviation or impact. In this study, we developed and internally validated the prediction model of ECV results. It is proved that our prediction model has a good ability to predict the success of ECV with a high C-index (0.841). It is superior to previous studies, such as 0.67 in Burgos et al.^47^, 0.70 in Kok et al.^48^, and 0.78 in Velzel et al.^49^. The outcome judgment of ECV is immediate, not at birth, which avoids the deviation of ECV outcome judgment. For example, the research of Morgan^27^ is based on the fetal position at birth, because they have no information about the immediate success after ECV, but only the information identified by the ICD-9 program code at birth, which may cause biased. We hope to modify the prediction model to a simpler scoring system to make it easier to be used by clinicians. Nomogram is such a tool, which can transform complex regression equations into simple and visual graphics, making the results of the prediction model more readable and more valuable. By observing the impact of each predictive variable on the outcome, clinicians can simply calculate the sum of all predictive effects for a given patient and predict the probability. In this study, we also confirmed the predictive value through both internal and external validations. We believe it will help disseminate knowledge and improve health care in the future. The limitations of this study are also worth mentioning. We recognize that although the internal validation results are ideal, our model still needs external validation to be used in clinical practice. To further confirm the universality of our model, we plan to externally validate the female group considering ECV and keep updating. In addition, the population in this cohort is mainly Chinese, which limits the applicability of our results to other ethnic environments. We also do not have long-term data on future pregnancy and childhood outcomes.

## Supplementary Information

Supplementary Information.

## Data Availability

The full trial protocol and all the data are available from the corresponding author, Ye Zhou (Email: zhouye_2022@126.com), upon reasonable request.
